# Translation and psychometric validation of the Chinese version of the metacognitive awareness scale among nursing students

**DOI:** 10.3389/fpsyg.2024.1354810

**Published:** 2024-05-16

**Authors:** Shasha Li, Jun Xu, Xuejing Jia, Yanjun Zhao, Xiaojing Liu, Yuecong Wang

**Affiliations:** ^1^Department of Nursing, College of Medical Science, Huzhou University, Huzhou, Zhejiang, China; ^2^Ningbo Municipal Hospital of Traditional Chinese Medicine (TCM), Affiliated Hospital of Zhejiang Chinese Medical University, Ningbo, Zhejiang, China; ^3^Hebei University of Chinese Medicine, Shijiazhuang, Hebei, China; ^4^Department of Nursing, Weifang University of Science and Technology, Shouguang, Shandong, China

**Keywords:** metacognitive awareness, nursing students, transcultural, reliability, validity

## Abstract

**Objective:**

This study endeavors to translate and psycho-metrically validate the metacognitive awareness inventory scale (MAS) for nursing students in China.

**Method:**

A total of 592 nursing students were enlisted from four universities situated in the eastern, southern, western, and northern regions of China. Content validity and reliability were evaluated using the content validity index and item-total correlation coefficient, and Cronbach’s alpha coefficients, respectively. Convergent validity examined the goodness of fit among sub-scales through the average extracted variance and composite reliability.

**Results:**

Exploratory factor analysis confirmed the first-order and second-order factor models, contributing to a cumulative variance of 89.4 and 59.5%, respectively. The Cronbach’s alpha values were 0.963 and 0.801, respectively. Confirmatory factor analysis outcomes indicated an excellent overall fit index for the model, satisfying the convergent validity criteria and achieving a target coefficient of 96.0%, which is consistent with the original scale structure.

**Conclusion:**

The Chinese version of the MAS (C-MAS) is a reliable and valid instrument for assessing metacognitive awareness among Chinese nursing students. Further research should consider a broader sample of nursing students across China to reinforce the scale’s applicability.

## 1 Introduction

The COVID-19 pandemic has led to a heightened demand for nursing professionals (Zabaleta-Del-Olmo et al., 2023). It is noteworthy that contemporary hospitals increasingly require nursing professionals to be adept at acquiring proficiency with new technologies to handle similar emergencies (Nascimento et al., 2023). In an era characterized by rapid advancements in medical technology, digital environments, and diverse patient needs, the development of metacognitive awareness among nursing professionals is crucial for improving work efficiency and fostering lifelong learning abilities (Breland et al., 2023). This underscores the demand for universities to cultivate metacognitive awareness among nursing students (Bektas et al., 2021).

Metacognitive awareness encompasses conscious, purposeful, and active cognitive processes, including thinking, planning, monitoring, adjustment, evaluation, and reflection on learning (González-Cabañes et al., 2022). These processes form the foundation for effective knowledge construction and monitoring. Previous studies have shown that metacognitive awareness can facilitate students in advancing their cognition, stimulating their interest in learning, enhancing their initiative, and optimizing their learning outcomes (Abdelrahman, 2020; Jung et al., 2022). It serves as a primary driver in improving students’ information retrieval abilities (Reisoglu and Toksoy, 2020), promoting collaborative learning, and facilitating the implementation of problem-solving strategies (Siqueira et al., 2020; Jin and Ji, 2021). Therefore, prioritizing the development of metacognitive awareness among nursing students is indispensable for their professional growth and development (Wolfe and Williams, 2018; Bektas et al., 2021).

Schraw and Dennison (1994) devised the Metacognitive Awareness Scale (MAS) to aid individuals and educational institutions in assessing the level of metacognitive awareness. This scale evaluates individuals across two dimensions: knowledge of cognition and regulation of cognition, thereby creating a multidimensional framework. Knowledge of cognition encompasses declarative knowledge, procedural knowledge, and conditional knowledge (González-Cabañes et al., 2022). Declarative knowledge involves understanding “about” concepts, procedural knowledge involves knowing “how” to perform tasks, and conditional knowledge encompasses the “why” and “when” aspects of cognition (Teo and Lee, 2012; Harrison and Vallin, 2018). Regulation of cognition includes planning, information management strategies, monitoring, debugging strategies, and evaluation, constituting a cyclical process of thought adjustment (Schraw and Dennison, 1994). These adjustments enable individuals to align internal demands with external environments, thus enhancing learning capabilities (Turan et al., 2009; Rivers et al., 2020).

While the Metacognitive Awareness Scale (MAS) has been translated and validated in various cultural contexts globally, including Turkey (Akin et al., 2007), Southeast Asian countries (Teo and Lee, 2012), Portugal (Lima-Filho and Bruni, 2015), the United States (Harrison and Vallin, 2018; Jung et al., 2022), and Spain (González-Cabañes et al., 2022), there remains a paucity of research on its application to assess metacognitive awareness among nursing undergraduate students in China. One significant limitation of the scale is the absence of a version that is validated for the Chinese cultural context, which impedes the study of metacognitive awareness among nursing students from diverse cultural backgrounds. Therefore, this study sought to evaluate the psychometric properties of the MAS within a Chinese context. These findings will contribute to a more comprehensive understanding of the construct of metacognitive awareness among nursing students.

## 2 Literature review

### 2.1 Nursing education and metacognitive awareness in China

Traditional nursing education in China has prioritized the development of students’ clinical operational skills, communication and coordination abilities, and critical thinking (Fu et al., 2022). However, there is a relative lack of emphasis on metacognitive awareness among nursing students (Pu et al., 2020). This oversight results in students’ deficiencies in cognitive processes such as learning planning, management, monitoring, adjustment, and evaluation, which in turn leads to a shortfall in their self-directed learning capabilities (Jin and Ji, 2021). In light of the rapidly changing landscape characterized by new technologies, digitalization, and automated educational environments, Chinese scholars (Sha et al., 2022) are working to bolster students’ metacognitive awareness through blended teaching reforms that integrate online and offline modalities in nursing practice training courses (Sha et al., 2022). These initiatives are designed to encourage the development of lifelong learning habits among nursing students.

In recent years, the advent of intelligent nursing services enabled by technologies such as cloud computing, big data, and the Internet of Things has led to heightened expectations for the intelligence, personalization, and precision of nursing education in China (Liu et al., 2023). The cultivation of lifelong learning among nursing students has become essential (Wong et al., 2021). Studies have shown that nursing students with elevated levels of metacognitive awareness are more capable of evaluating their learning situations, planning learning actions, and adjusting learning strategies, which in turn improves learning efficiency and promotes the development of positive study habits (Desender et al., 2021; Du et al., 2023). Nursing students who possess higher levels of metacognitive awareness can better understand and assimilate knowledge across various disciplines, supporting the establishment of lifelong learning habits and the ongoing enhancement of professional knowledge and skillsets to meet the evolving demands of the medical landscape (Gholami et al., 2016).

Currently, despite the integration of virtual simulation teaching, high-quality online classrooms, and other training modalities to augment nursing students’ metacognitive abilities, there remains a paucity in the depth of analysis and discourse regarding the content of metacognitive awareness within nursing education (Barranquero-Herbosa et al., 2022). As a result, there is an imperative need for an accurate assessment tool to gauge the efficacy of metacognitive awareness training among nursing students. Such a tool is crucial for establishing a theoretical framework that can inform the development of more precise educational strategies aimed at enhancing nursing students’ metacognitive awareness.

### 2.2 Measuring metacognitive awareness in the previous

In China, three prevailing scales have been employed to measure metacognitive awareness among Chinese nursing students: the Metacognitive Ability Scale for College Students, developed by Chinese scholar Kang Ye et al. (2018), the Metacognitive Inventory for Nursing Students by Hsu (2010), and the State Metacognitive Inventory by O’Neil and Abedi (1996). These instruments assess metacognitive abilities directly, offering an indirect reflection of changes in metacognitive awareness through these abilities. However, they do not directly investigate and elucidate the multifaceted dimensions of metacognitive awareness specific to nursing students. Consequently, there is a need for research to employ internationally recognized metacognitive awareness scales and to conduct psychometric evaluations within the Chinese linguistic context to ascertain their validity. Adopting this method will facilitate precise monitoring of fluctuations in nursing students’ metacognitive awareness, providing a robust theoretical framework for comparative studies on metacognitive awareness among students from varying cultural backgrounds, thereby enriching cross-cultural inquiry in the field of nursing education.

## 3 Methods

### 3.1 Study design

Cross-cultural adaptation and validation of C-MAS were conducted in a methodological cross-sectional study (Huang et al., 2020).

### 3.2 Participants

In adherence to the standard guidelines for sample size (Myers et al., 2011), which recommend a ratio of at least 5:1 between sample size and parameters, the study was conducted with 52 items in mind. To account for potential invalid samples, the sample size was increased by 15%. Consequently, the required sample size was determined to be 299, ensuring it met the maximum threshold.

Study participants were recruited through convenient sampling from four universities in eastern, southern, western, and northern China between January and July 2022. The inclusion criteria were: (i) full-time nursing undergraduates and (ii) willingness to provide informed consent for participation. The exclusion criterion was students pursuing a degree upgrade from a junior college to a bachelor’s level. The study was approved by the Ethics Committee of Huzhou University (Approval No.: 202012-JG02), with voluntary participation and confidentiality assured. A total of 592 nursing undergraduates completed the survey, with an average age of 21.24 years (standard deviation [SD], 1.65). The sample comprised 512 females (86.5%) and 80 males (13.5%). In terms of academic standing, 142 (24.0%) were freshmen, 140 (23.6%) were sophomores, 186 (31.4%) were juniors, and 124 (20.9%) were seniors.

### 3.3 Instrument

#### 3.3.1 General condition questionnaire

The demographic questionnaires included questions about gender, age, and grade.

#### 3.3.2 Metacognitive awareness scale

The Metacognitive Awareness Scale (MAS) is a 52-item self-report instrument that encompasses two overarching factors: ‘Knowledge of Cognition’ (17 items) and ‘Regulation of Cognition’ (35 items). A detailed structural description can be found in Table 1. The scale utilizes a 5-point Likert scale, with scores ranging from 1 (always false) to 5 (always true). An average score of 2.5 for each item represents a mid-level score, with scores above 2.5 indicating performance exceeding the mid-level. The total score spans from 52 to 260, with higher scores denoting a greater awareness of cognition and its regulation. The coefficient alpha (α) for items loading onto each factor is 0.91, and the full scale has an overall Cronbach’s alpha coefficient of 0.95, signifying high internal consistency (Schraw and Dennison, 1994).

**Table 1:** Operational definitions of content included in the MAS and items.

Categories	Subcategories	Definition	Quantity	Items
Knowledge of cognition	Declarative knowledge (DK)	Knowledge of one’s skills, intellectual resources, and abilities as a learner	8	5, 10, 12, 16, 17, 20, 32, 46
	Procedural knowledge (PK)	Knowledge of how to implement learning procedures	4	3,14, 27, 33
	Conceptual knowledge (CK)	Knowledge of when and why to use learning procedures	5	15, 18, 26, 29, 35
Regulation of cognition	Planning (P)	Planning, goal setting, and allocating resources prior to learning	7	4, 6, 8, 22, 23, 42, 45
	Information management strategies (IMS)	Skills and strategy sequences used online to process information more efficiently (e.g., organizing, elaborating, summarizing, and selective focusing)	10	9, 13, 30, 31, 37, 39, 41, 43, 47, 48
	Monitoring (M)	Assessment of one’s learning or strategy use	7	1, 2, 11, 21, 28, 34, 49
	Debugging strategies (DS)	Strategies used to correct comprehension and performance errors	5	25, 40, 44, 51, 52
	Evaluation (E)	Analysis of performance and strategy effectiveness after a learning episode	6	7, 19, 24, 36, 38, 50

### 3.4 Psychometric testing procedures

The translation and adaptation process of the MAS was based on Brislin’s translation model (Jones et al., 2001), following the two phases below.

Phase I comprised four steps. (i) Forward translation: the scale was independently translated into Chinese by two researchers proficient in English and Chinese. Subsequently, the researchers evaluated the congruity between the two translated versions through discussions with the research team, leading to the development of the final version. (ii) Back translation: two non-nursing researchers independently translated the scale back into English. One researcher held a doctoral degree in English translation, while the other possessed 10 years of experience in metacognition research and education in an English-speaking country. The two translated versions were compared, and their similarities and differences were analyzed. (iii) Cultural adaptation: a panel of nine experts reviewed the concept and connotation of the translated original scale, and scored the content relevance. This expert panel included two professors with expertise in psychological nursing, two professors specializing in nursing education management, three teachers with over 15 years of metacognition research, and two frontline nurses with more than 10 years of clinical experience. Leveraging their professional theoretical knowledge, educational background, and clinical expertise, the experts independently assessed each scale item for clarity of expression, conceptual equivalence, and content relevance. Each item was evaluated individually, and any ambiguous aspects were revised accordingly. (iv) Content validity: content validity was determined through expert scoring (Polit and Beck, 2014), with the above-mentioned nine experts participating in the content validity review.

Phase II comprised two steps. (i) A convenience sampling approach was used to enlist 30 nursing students for the evaluation of their understanding of the Chinese version of the Metacognitive Awareness Scale (C-MAS). The goal was to ensure that the scale could be easily understood by Mandarin-speaking nursing students. Participants were asked if they needed assistance understanding and responding to the questionnaire items. All items were fully understood, and participants completed the questionnaire without issue. (ii) Utilizing factor analysis to examine the factorial structure of the Chinese version of the metacognitive awareness scale. A total of 592 nursing students were recruited, and the samples were randomly divided into Group A and Group B based on matching criteria such as grade and gender. Data from Group A were subjected to exploratory factor analysis (EFA), while data from Group B underwent confirmatory factor analysis (CFA). Each group consisted of 296 nursing students.

### 3.5 Data collection

The study received support from the management department of the surveyed school. The researcher provided a detailed explanation of the study’s purpose and significance to the participants, and each participant signed an informed consent form. Paper versions of the questionnaire were distributed in sealed envelopes to the participants in their classrooms. Participants were asked to answer the questionnaire on the spot and return it immediately. The completeness of each questionnaire was checked, and any missing items were addressed by asking the participants again and supplementing the information promptly. The researchers confirmed that all answers were complete. To ensure the completeness and authenticity of the responses, four researchers, uniformly trained, were responsible for distributing and collecting the survey questionnaires. The targeted sample size was 598 nursing students, and a total of 592 valid questionnaires met the criteria, resulting in a valid response rate of 98.99%.

### 3.6 Data analysis

Statistical analysis of the collected data was performed using IBM SPSS 22.0 and Mplus software version 7.4 (Muthen and Muthen, Los Angeles, CA, United States). Descriptive statistics, including frequencies and percentages, were utilized to summarize the characteristics of the respondents’ basic information. The reliability of the C-MAS was evaluated through item-total correlation coefficients and internal consistency, as measured by Cronbach’s alpha coefficients.

Construct validity was assessed through exploratory factor analysis (EFA) and confirmatory factor analysis (CFA). EFA was employed to explore the factor model of the C-MAS, utilizing principal component analysis and the maximum variance method. The sampling adequacy was determined using the Kaiser–Meyer–Olkin (KMO) measure, with a KMO value exceeding 0.8, and Bartlett’s sphericity test showing a *p-*value less than 0.01. Furthermore, additional criteria were applied to refine the factor structure and determine the optimal number of factors: eigenvalues greater than 1.0, factor loadings exceeding 0.50, and a percentage of total variance explained (Hou et al., 2004; Zhao et al., 2023).

The CFA was used to confirm the prior explored model obtained through EFA. Model fit was assessed using the following indices: (a) Chi-square/degrees of freedom (χ^2^/df) ratio, ideally falling between 1 and 3. (b) Root mean square error of approximation (RMSEA) with values less than 0.08. (c) Standardized root mean square residual (SRMR) values less than 0.06, and RMSEA with a 90% confidence interval (RMSEA 90%CI). (d) Normed fit index (NFI), incremental fit index (IFI), and goodness of fit index (GFI) with values greater than 0.90 (Zhao et al., 2023).

Convergent validity was evaluated by examining the fit of the subscales using average variance extracted (AVE) and composite reliability (CR). An AVE exceeding 0.5 is considered satisfactory, while a CR surpassing 0.6 is deemed acceptable, both indicating sound structural reliability (Xue and Zhang, 2017). In line with the methodology proposed by Marsh and Hocevar (1985), two models were identified: an 8-factor first-order model and an 8-factor second-order model, to assess their adequacy. A target coefficient value approximating 1 suggests that the second-order model could effectively substitute the first-order model, thus enhancing the overall precision of the model.

Content validity index (CVI) was calculated to assess content validity. Nine experts participated in the validity analysis, with each expert providing a score for the relevance of each item on a scale of 1 (not relevant) to 4 (definitely relevant). An item is considered valid if its content validity score is 0.80 or higher (Luo et al., 2019).

## 4 Results

### 4.1 Item analysis

The findings revealed that the average score for each item measuring metacognitive awareness among nursing students surpassed 2.50. This implies that the overall level of metacognitive awareness was above the midpoint, as detailed in Table 2. Furthermore, each item was subjected to an item-total correlation test. The outcomes showed that the scores for each item were significantly and positively correlated with the total scale score, with all correlations exceeding 0.4, suggesting the absence of multicollinearity. Additionally, every item achieved statistical significance (*p* < 0.05). Consequently, all 52 items fulfilled the pre-established criteria and were retained, with no items being removed.

**Table 2:** Content validity indexes, means, SDs, and corrected item total correlations for the C-MAS (*n* = 296).

Item		Mean	*SD*	Item total correlation	Item		Mean	*SD*	Item total correlation
DK1	I5	3.30	0.77	0.527^*^	IMS3	I30	3.31	0.76	0.737^*^
DK2	I10	3.28	0.83	0.512^*^	IMS4	I31	3.33	0.77	0.742^*^
DK3	I12	3.32	0.79	0.526^*^	IMS5	I37	3.31	0.76	0.738^*^
DK4	I16	3.31	0.82	0.550^*^	IMS6	I39	3.3	0.78	0.736^*^
DK5	I17	3.26	0.77	0.502^*^	IMS7	I41	3.31	0.75	0.724^*^
DK6	I20	3.31	0.78	0.524^*^	IMS8	I43	3.32	0.78	0.776^*^
DK7	I32	3.28	0.75	0.506^*^	IMS9	I47	3.35	0.76	0.763^*^
DK8	I46	3.27	0.76	0.467^*^	IMS10	I48	3.31	0.73	0.727^*^
PK1	I3	3.11	0.85	0.553^*^	M1	I1	3.30	0.75	0.716^*^
PK2	I4	3.10	0.88	0.509^*^	M2	I2	3.30	0.75	0.695^*^
PK3	I27	3.13	0.88	0.565^*^	M3	I11	3.29	0.74	0.690^*^
PK4	I33	3.08	0.83	0.491^*^	M4	I21	3.29	0.75	0.678^*^
CK1	I15	3.30	0.70	0.489^*^	M5	I28	3.29	0.74	0.680^*^
CK2	I18	3.31	0.74	0.490^*^	M6	I34	3.28	0.73	0.690^*^
CK3	I26	3.36	0.71	0.505^*^	M7	I49	3.29	0.76	0.707^*^
CK4	I29	3.35	0.74	0.494^*^	DS1	I25	3.44	0.76	0.525^*^
CK5	I35	3.37	0.77	0.482^*^	DS2	I40	3.46	0.74	0.572^*^
P1	I4	3.19	0.76	0.689^*^	DS3	I44	3.45	0.75	0.557^*^
P2	I6	3.19	0.77	0.685^*^	DS4	I51	3.46	0.75	0.582^*^
P3	I8	3.21	0.78	0.698^*^	DS5	I52	3.44	0.70	0.547^*^
P4	I22	3.22	0.80	0.712^*^	E1	I7	3.34	0.78	0.638^*^
P5	I23	3.22	0.80	0.706^*^	E2	I19	3.30	0.79	0.546^*^
P6	I42	3.19	0.77	0.695^*^	E3	I24	3.36	0.80	0.661^*^
P7	I45	3.20	0.77	0.709^*^	E4	I36	3.36	0.80	0.661^*^
IMS1	I9	3.32	0.79	0.735^*^	E5	I38	3.35	0.80	0.634^*^
IMS2	I13	3.31	0.78	0.757^*^	E6	I50	3.36	0.81	0.653^*^

^*^p < 0.05; the middle level of the Mean-value is 2.5; I1 ~ I52 denote Item1 ~ Item52.

### 4.2 Reliability and validity analysis

#### 4.2.1 Exploratory factor analysis

The EFA was conducted to investigate the initial structure of the C-WAS. The KMO measure yielded a value of 0.841, and Bartlett’s test of sphericity showed a chi-square value of 34840.65 (*p* < 0.001), indicating the data were suitable for exploratory factor analysis. Eight factors were extracted with eigenvalues ranging from 2.977 to 9.587, and the items’ factor loadings ranged from 0.687 to 0.939. Together, these factors explained 89.445% of the overall variance.

Additionally, the analysis of the second-order factor revealed a KMO measure of 0.811, and Bartlett’s test of sphericity had a chi-square value of 701.62 (*p* < 0.001), indicating the second-order factor data were suitable for exploratory factor analysis. Eight factors were extracted with eigenvalues ranging from 2.062 to 2.697, and the items’ factor loadings ranged from 0.605 to 0.815. Collectively, these factors explained 59.497% of the overall variance.

The 52 items of the MAS were categorized into eight factors, forming a first-order model. The results of the factor analysis indicated that the Cronbach’s alpha coefficient for the first-order model of C-WAS was 0.963, with factors ranging from 0.875 to 0.992. These findings suggest good reliability, reflecting satisfactory internal consistency within the first-order Model C-WAS. Subsequently, the eight factors of the metacognitive awareness scale were further grouped into two sub-dimensions, creating a second-order model. The results of the factor analysis revealed that the Cronbach’s alpha coefficient for the C-WAS was 0.800, with factors measuring at 0.756 and 0.788. These results also indicated good reliability, signifying satisfactory internal consistency within the second-order model C-WAS, as outlined in Table 3.

**Table 3:** Exploratory factor analysis of first- and second-order results of the C-WAS (*n* = 296).

Items		First-order factors								Second-order factors	
		DK	PK	CK	P	IMS	M	DS	E	KC	RC
Factor 1: Declarative knowledge (DK)	I5	0.906									
	I10	0.896									
	I12	0.894									
	I16	0.892									
	I17	0.888									
	I20	0.870									
	I32	0.853									
	I46	0.839									
Factor 2: Procedural knowledge (PK)	I3		0.851								
	I4		0.847								
	I27		0.750								
	I33		0.780								
Factor 3: Conceptual knowledge (CK)	I15			0.751							
	I18			0.748							
	I26			0.745							
	I29			0.722							
	I35			0.687							
Factor 4: Planning (P)	I4				0.893						
	I6				0.887						
	I8				0.886						
	I22				0.880						
	I23				0.876						
	I42				0.876						
	I45				0.872						
Factor 5: Information management strategies (IMS)	I9					0.910					
	I13					0.907					
	I30					0.906					
	I31					0.901					
	I37					0.898					
	I39					0.894					
	I41					0.891					
	I43					0.889					
	I47					0.886					
	I48					0.850					
Factor 6: Monitoring (M)	I1						0.876				
	I2						0.872				
	I11						0.870				
	I21						0.867				
	I28						0.865				
	I34						0.839				
	I49						0.816				
Factor 7: Debugging strategies (DS)	I25							0.939			
	I40							0.935			
	I44							0.929			
	I51							0.923			
	I52							0.917			
Factor 8: Evaluation (E)	I7								0.929		
	I19								0.920		
	I24								0.919		
	I36								0.920		
	I38								0.917		
	I50								0.900		
DK total score										0.815	
CK total score										0.782	
P total score											0.807
IMS total score											0.774
M total score											0.747
Ds total score											0.642
E total score											0.605
Common factors with eigenvalue		9.587	7.072	6.546	6.307	5.790	4.876	3.356	2.977	2.062	2.697
Cumulative variance contribution rate		18.437	32.037	44.625	56.754	67.889	77.265	83.719	89.445	25.781	59.497
α coefficients of each dimension		0.992	0.972	0.991	0.986	0.992	0.991	0.875	0.915	0.756	0.788

I represents item number. The first-order factor includes 8 factor, the second-order factor includes 2 sub-variables, i.e., KC. knowledge of cognition; RC, regulation of cognition.

#### 4.2.2 Confirmatory factor analysis

Based on the 52 items retained from the EFA, a CFA (*N* = 296, Group B) was conducted to examine the first- and second-order models. The first-order model exhibited an acceptable fit (*χ*^2^ = 2194.63, df = 1,265, *χ*^2^/df = 1.73, CFI = 0.939, TLI = 0.936, SRMR = 0.047). Building upon this, the study further validated the second-order model, and the model indices were found to be acceptable (*χ*^2^ = 2106.88, df = 1,246, *χ*^2^/df = 1.69, CFI = 0.943, TLI = 0.939, SRMR = 0.032), as detailed in Table 4.

**Table 4:** Model fitting results (*N* = 296).

Model	Factors	CRs	AVEs	Model fitting results	Target coefficient
Fist-order model	DK	0.918	0.587	*X*^2^ = 2194.63*, df = 1,265, *X*^2^/df = 1.73,CFI = 0.939,TLI = 0.936, SRMR = 0.047, RMSEA(90%CI) = 0.050(0.045,0.052)	96.00%
	PK	0.893	0.676		
	CK	0.868	0.570		
	P	0.946	0.713		
	IMS	0.962	0.719		
	M	0.946	0.716		
	DS	0.927	0.719		
	E	0.922	0.664		
Second-order model	KC	0.894	0.738	*X*^2^ = 2106.88^*^,df = 1,246, *X*^2^/df = 1.69, CFI = 0.943,TLI = 0.939,SRMR = 0.032, RMSEA(90%CI) = 0.048(0.046,0.053)	
	PC	0.989	0.950		

*p < 0.01; X^2^/df, chi-square/degrees of freedom; TLI, Tucker–Lewis index; CFI, comparative fit index; CR, combination reliability. SRMR, standardized root mean square residual; RMSEA (90% CI), root mean square error of approximation with 90% confidence interval; AVE, average variance extracted. Target coefficient, Second-order model’s RMSEA(90%CI)/Fist-order model’s RMSEA(90%CI).

**Figure 1:**
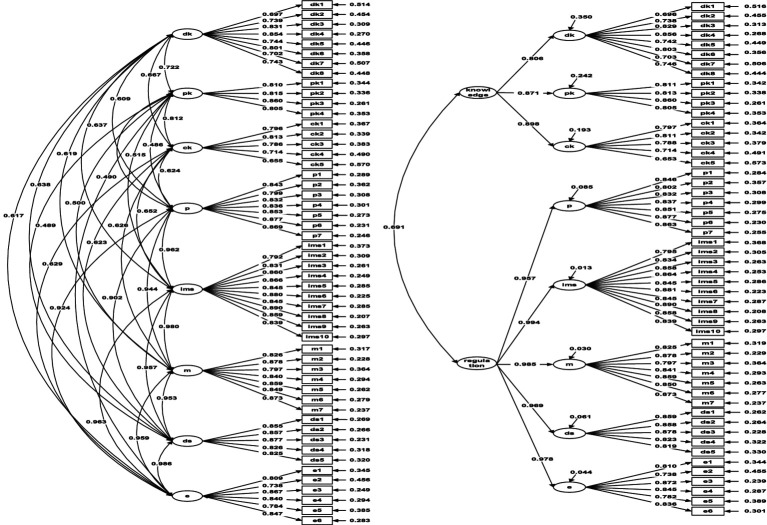
First-order and Second-order model of the C-MAI. Analysis using Mplus 8.0 (*n* = 296); dk, declarative knowledge; pk, procedural knowledge; ck, conceptual knowledge; p, planning; ims, information management strategies; m, monitoring; ds, debugging strategies; e, evaluation. Knowledge, knowledge of cognition; recognition, regulation of cognition.

For further analysis of convergent validity, Table 4 illustrates that the first-order model’s eight factors have AVE ranging from 0.587 to 0.791 and CR from 0.868 to 0.962. Meanwhile, the second-order model demonstrates AVE between 0.894 and 0.989, and CR between 0.738 and 0.962. Additionally, the target coefficient is 96%, suggesting that the second-order model can effectively replace the first-order model, enhancing precision, as depicted in Figure 1.

### 4.3 Content validity

Nine experts, including professionals from the fields of psychiatric nursing, frontline clinical nursing, nursing education management, and research related to metacognition, evaluated the relevance of each item to metacognitive awareness. Employing a Likert 4-point rating scale, where 4 points denoted high relevance, 3 points indicated moderate relevance, 2 points suggested low relevance, and 1 point reflected irrelevance. The content validity index at the item level was calculated based on the ratings provided by the nine experts. The results indicated that the Content Validity Index at the scale level (S-CVI) was 0.912, and the Content Validity Index at the item level (I-CVI) ranged from 0.855 to 1.

## 5 Discussion

Despite the crucial importance of metacognitive awareness in exploring students’ active learning mechanisms, there is currently a lack of comprehensive assessment tools for studying metacognitive awareness among nursing students (Pu et al., 2020). To the best of our knowledge, this is the first scale available for investigating metacognitive awareness among nursing students in mainland China. This scale lays the groundwork for conducting large-scale studies, gaining a deeper understanding of the metacognitive awareness status among nursing students, and providing essential information for cross-national comparisons of metacognitive awareness among nursing students (Pour and Ghanizadeh, 2017; Rashwan et al., 2021).

Due to the differences in natural and social conditions between the East and the West, cognitive knowledge acquisition and cognitive adjustment often diverge in habitual thinking and language expression (Desender et al., 2021; Martirosov and Moser, 2021). To achieve cross-cultural adaptation and equivalence in translation, we consulted with experts and attempted to translate the scale into Chinese using simplified vocabulary. After expert consultation, item 15 (“I learn best when I know something about the topic”) and item 48 (“I focus on the overall measurement rather than the norm”) were revised to item 15 (“I am more willing to learn when I know about a topic”) and item 48 (“I focus on overall concepts and details”). The revised item content aligns more closely with the understanding of nursing students in the Chinese cultural context.

Our results demonstrate that the 52-item Chinese version of the Metacognitive Awareness Scale (C-WAS) is an effective and reliable tool with satisfactory content validity, acceptable internal consistency, and commendable construct validity. The C-WAS effectively captures metacognitive awareness in Chinese nursing undergraduates. Conversely, this contrasts with findings from a study involving Spanish university students that utilized a concise metacognitive awareness tool comprising 19 items (González-Cabañes et al., 2022). The discrepancy may stem from variations in cultural beliefs, educational approaches, and learning contexts among international student populations (Bektas et al., 2021). Emerging research suggests that a comprehensive assessment of metacognitive awareness necessitates an exhaustive examination of the scope and attributes of each variable (Yu et al., 2023). To gauge the evolution of metacognitive awareness in nursing students, scholars should prioritize unraveling the significance of items related to cognitive knowledge and cognitive regulation (Abdelrahman, 2020; Buratin, 2024), corroborating the outcomes of our current study.

However, concerning reliability, the first- and second-order C-WAS models demonstrated robust reliability and internal consistency. The content validity indices, including the Item-Content Validity Index (I-CVI) scores and the Scale-Content Validity Index (S-CVI) scores, were both above 0.80, indicating that the C-WAS possesses strong content validity. Exploratory factor analysis (EFA) results revealed that each factor loading was greater than 0.5, and the factor structure explained 59.5% of the total variance, exceeding the 50.0% threshold. Confirmatory factor analysis (CFA) outcomes indicated an excellent overall fit for both the second-order and first-order factor models. These findings substantiate that the 8-factor first-order model (encompassing declarative knowledge, procedural knowledge, conceptual knowledge, planning, information management strategies, monitoring, debugging strategies, and evaluation) exhibits commendable reliability and validity. Furthermore, the two higher-order sub-variables, namely knowledge of cognition and regulation of cognition, also demonstrate robust reliability and validity.

Additional analysis from further studies revealed that the Composite Reliability (CR) surpassed the Average Variance Extracted (AVE), and the AVE exceeded 0.5, suggesting a strong convergence validity among the subscales (Buratin, 2021). The target coefficient for comparing the first-order and second-order factor models was 96.0%. This indicates that the second-order factor model can effectively elucidate the intergroup associations with the first-order factors model, endorsing the existence of two higher-order sub-variables: knowledge of cognition and regulation of cognition. Consequently, our results corroborate the original scale’s findings, confirming that the C-WAS scale comprises two sub-dimensions, eight factors, and 52 items, demonstrating robust construct validity.

Cross-cultural translation and psychometric evaluation enable a deeper scientific understanding of metacognitive consciousness. This process lays the groundwork for investigating metacognitive awareness across various domains and professions (Siqueira et al., 2020). Our findings align with the research outcomes of Harrison and Vallin (2018), underscoring the importance of elaborating upon and quantifying cognitive knowledge and cognitive regulation in crafting precise educational intervention plans and strategies (Rivers et al., 2020; Lovell et al., 2021). Future investigations may employ the C-MAS to assess the metacognitive awareness of nursing students across different regions in China, providing an essential tool for comparative studies on students’ metacognitive awareness within diverse cultural settings. These enhance our understanding of the differences and similarities among various cohorts. Moreover, utilizing the C-MAS as an effective evaluation instrument can inform the development and refinement of educational programs concerning metacognitive skills (Siena and Simons, 2024). Researchers can assess nursing students’ metacognitive awareness accurately and impartially, supporting the development and implementation of educational strategies.

## 6 Limitation

Three limitations need to be pointed out. Firstly, the outcomes of this investigation reveal higher alpha coefficients. High alpha values may suggest that the scales are excessively lengthy, comprise redundant items, or offer limited coverage of the construct being measured (Panayides, 2013). Thus, future research should endeavor to assess the multidimensional scale with an expanded sample size. Secondly, the sample population was confined to undergraduate nursing students from four universities in China, thus necessitating further psychometric evaluation of the scale using a more diverse sample to confirm its reliability and validity for application among undergraduate nursing students across various regions. Thirdly, the study may be subject to social desirability bias. Despite the voluntary and anonymous nature of participation in the questionnaire, nursing students, influenced by their specific cultural context, might subconsciously conform to expected learning behaviors and present a positive self-image.

## 7 Conclusion

This study conducted a cross-cultural debugging of the MAS and preliminary psychometric measurements and revisions for undergraduate nursing students to form a Chinese version of the C-MAS scale with acceptable reliability and validity. This instrument can be utilized to assess the metacognitive awareness levels of undergraduate nursing students, highlighting the importance of cognitive knowledge and cognitive regulation among this population. It offers a theoretical benchmark for the creation of intervention strategies tailored to enhance metacognitive skills in nursing education.

## Data availability statement

The data that support the findings of this study are available from the corresponding author upon reasonable request. Requests to access these datasets should be directed to 270037615@qq.com.

## Ethics statement

This study was reviewed by the Ethics Committee of Huzhou University (Number: 202012-JG02). The studies were conducted in accordance with the local legislation and institutional requirements. The participants provided their written informed consent to participate in this study.

## Author contributions

SL: Writing – original draft, Writing – review & editing. JX: Data curation, Writing – review & editing. XJ: Formal analysis, Investigation, Writing – review & editing. YZ: Data curation, Validation, Writing – review & editing. XL: Investigation, Methodology, Visualization, Writing – review & editing. YW: Investigation, Supervision, Software, Writing – review & editing.
